# Skeletal Muscle Transcriptome Analysis of Hanzhong Ma Duck at Different Growth Stages Using RNA-Seq

**DOI:** 10.3390/biom11020315

**Published:** 2021-02-19

**Authors:** Zhigang Hu, Junting Cao, Jianqin Zhang, Liyan Ge, Huilin Zhang, Xiaolin Liu

**Affiliations:** College of Animal Science and Technology, Northwest A&F University, Yangling 712100, Shaanxi, China; huzg2017060@163.com (Z.H.); fightingcaoting@nwafu.edu.cn (J.C.); zhangjianqin1356@126.com (J.Z.); a13149600612@163.com (L.G.); zhhl7461@nwsuaf.edu.cn (H.Z.)

**Keywords:** Hanzhong Ma duck, skeletal muscle, transcriptome, DEG, KEGG pathway

## Abstract

As one of the most important poultry worldwide, ducks (*Anas platyrhynchos*) are raised mainly for meat and egg products, and muscle development in ducks is important for meat production. Therefore, an investigation of gene expression in duck skeletal muscle would significantly contribute to our understanding of muscle development. In this study, twenty-four cDNA libraries were constructed from breast and leg muscles of Hanzhong Ma ducks at day 17, 21, 27 of the embryo and postnatal at 6-month-old. High-throughput sequencing and bioinformatics were used to determine the abundances and characteristics of transcripts. A total of 632,172,628 (average 52,681,052) and 637,213,938 (average 53,101,162) reads were obtained from the sequencing data of breast and leg muscles, respectively. Over 71.63% and 77.36% of the reads could be mapped to the *Anas platyrhynchos* genome. In the skeletal muscle of Hanzhong duck, intron variant (INTRON), synonymous variant (SYNONYMOUS_CODING), and prime 3′ UTR variant (UTR_3_PRIME) were the main single nucleotide polymorphisms (SNP) annotation information, and “INTRON”, “UTR_3_PRIME”, and downstream-gene variant (DOWNSTREAM) were the main insertion-deletion (InDel) annotation information. The predicted number of alternative splicing (AS) in all samples were mainly alternative 5′ first exon (transcription start site)-the first exon splicing (TSS) and alternative 3′ last exon (transcription terminal site)-the last exon splicing (TTS). Besides, there were 292 to 2801 annotated differentially expressed genes (DEGs) in breast muscle and 304 to 1950 annotated DEGs in leg muscle from different databases. It is worth noting that 75 DEGs in breast muscle and 49 DEGs in leg muscle were co-expressed at all developmental points of comparison, respectively. The RNA-Seq data were confirmed to be reliable by qPCR. The identified DEGs, such as *CREBL*2, *RHEB*, *GDF*6, *SHISA*2, *MYLK*2, *ACTN*3, *RYR*3, and *STMN*1, were specially highlighted, indicating their strong associations with muscle development in the Hanzhong Ma duck. KEGG pathway analysis suggested that regulation of actin cytoskeleton, oxidative phosphorylation, and focal adhesion were involved in the development of skeletal muscle. The findings from this study can contribute to future investigations of the growth and development mechanism in duck skeletal muscle.

## 1. Introduction

Skeletal muscle has a primary function of locomotion and protection, and it is also responsible for the structure and metabolic regulation of the body [1]. Besides, skeletal muscle mass represents up to 40% of total body weight in animals, which is an important trait in poultry breeding due to its high economic value [2,3]. In recent years, there have been many studies on the muscle development of poultry embryos [4]. The mechanism of myofibers formation involves the activation of progenitor cells, which proliferate as mononuclear myoblasts and eventually fuse to form multinucleated myotubes. Besides, many genes, non-coding RNAs, and transcription factors are involved in the regulation of muscle proliferation and differentiation [5,6]. Some studies have analyzed the gene expression changes involved in the muscle development of poultry [7,8]. However, the molecular mechanism of skeletal muscle development currently remains unclear.

RNA-Seq is a useful tool to measure gene transcription and better understand the physiology behind specific phenotypes. In the past few years, transcriptome sequencing has been applied to livestock and poultry and helped to select candidate genes related to important traits by comparing the global gene expression profiles between different animal populations in specific traits [9,10,11]. So far, RNA-Seq has been used to find and study the specific genes and pathways of muscle development under different conditions including cattle [12], sheep [13], pig [14], chicken [15], duck [16], and goose [17]. Hanzhong Ma duck is a local duck breed in China, which is mainly distributed in Hanzhong City, Shaanxi Province, China. It is small in size and light in weight. The average weight of male and female adult Hanzhong Ma ducks is 1.172 kg and 1.238 kg respectively, which greatly limits their meat value. Moreover, comparative transcriptomic studies on breast and leg muscle of Hanzhong Ma duck at different growth stages have been scarce.

In this study, the skeletal muscle transcriptomes of Hanzhong Ma duck at different growth stages were compared using RNA-Seq technology, and single nucleotide polymorphisms (SNP), InDel, and alternative splicing (AS) were detected. Besides, the functions of these differentially expressed genes (DEGs) were annotated and analyzed by the Gene Ontology (GO) and Kyoto Encyclopedia of Genes and Genomes (KEGG) database in order to identify the genes and genetic pathways related to the development of skeletal muscle in Hanzhong Ma ducks. The purpose of this study is to understand the genetic basis of breast and leg muscle development in ducks at the transcriptome level and to provide new insights into skeletal muscle development in poultry.

## 2. Materials and Methods

### 2.1. Animal and Tissue Collection

After disinfection, one hundred eggs of Hanzhong Ma ducks (HZ) were incubated in a standard incubator. On 17d (E17), 21d (E21), and 27d (E27) of incubation, 8 eggs were randomly selected to collect breast and leg muscles for DNA and RNA extraction. The DNA of duck muscle was amplified to determine the sex of embryonic ducks using gCHD primers [18], and the identified female embryos were selected as the research objects (Table 1). Besides, 6-month-old female ducks (M6), raised under the same environmental conditions with free access to water and commercial corn-soybean-based diets (Table S1), were slaughtered quickly to separate breast and leg muscles. The separated muscle tissues were immediately frozen in liquid nitrogen and then stored at −80 °C until use. Animal care, slaughter, and experimental procedures were approved by the Institutional Animal Care and Institutional Ethics Committee of Northwest A&F University (ethic code: #0330/2019), and all efforts were made to minimize suffering.

### 2.2. Library Construction and Sequencing

Total RNA was extracted using the QIAzol Lysis Reagent (QIAGEN, Berlin, Germany) following the manufacturer’s protocol. The OD260/280 and OD260/230 were measured to confirm the purity of the RNA sample, and the ranges were 1.8–2.0 and >2.0, respectively. The RNA purity was verified by agarose gel electrophoresis, and the RNA integrity was detected using the Bioanalyzer 2100 system (Agilent Technologies, San Jose, USA) with a minimum RNA integrity number (RIN) value of 7.3 (Table S2). Twenty-four sequencing libraries were made using the TruSeq PE Cluster Kit v4-cBot-HS (Illumia, San Diego, CA, USA)) according to the manufacturer’s protocol. The libraries were sequenced on the Illumina platform (Illumina HiSeq X Ten platform, San Diego, CA, USA) to produce 150 bp paired-end reads.

### 2.3. Mapping of the Reads to Reference Genome

By discarding reads containing adapters or poly-N and more than 10% of unknown nucleotides, and rejecting reads with more than 50% of low-quality (Q-value ≤ 10) bases, the high-quality clean data were obtained for subsequent analyses. Then the quality parameters for filtered data including Q30, GC content, and sequence-duplication level were calculated. The filtered reads were mapped to the *Anas platyrhynchos* genome sequence (https://www.ncbi.nlm.nih.gov/genome/?term=DUCK; Accessed on: 27 December 2019) and annotated transcripts (https://www.ncbi.nlm.nih.gov/assembly/GCF_003850225.1; Accessed on: 27 December 2019), and the unmapped or multi-position matched reads (≥2 mismatch reads) were excluded from further analyses. A duck genome map was drawn by using the HISAT 2 tool (http://ccb.jhu.edu/software/hisat2/index.shtml; Accessed on: 27 December 2019).

### 2.4. Analysis of SNP/InDel

According to the results of the HISAT 2 comparison between the reads and the *Anas platyrhynchos* genome sequence, the potential SNP sites and the InDels were identified and detected by the GATK software (GATK2, Broad Institute, Cambridge, MA, USA, https://software.broadinstitute.org/gatk/; Accessed on: 30 December 2019).

### 2.5. Identification of AS Events

AS events were identified, and the types of AS corresponding expression in each sample were obtained by using the ASprofile software (ASprofile, The Center for Computational Biology at Johns Hopkins University, Washington, USA) (http://ccb.jhu.edu/software/ASprofile/; Accessed on: 30 December 2019). There were 12 main types of AS events in duck skeletal muscle: (A) TSS: Alternative 5′ first exon (transcription start site) the first exon splicing; (B) TTS: Alternative 3′ last exon (transcription terminal site) the last exon splicing; (C) SKIP: Skipped exon single exon skipping; (D) XSKIP: Approximate SKIP single exon skipping (fuzzy boundary); (E) MSKIP: Multi-exon SKIP multi-exon skipping; (F) XMSKIP: Approximate MSKIP multi-exon skipping (fuzzy boundary); (G) IR: Intron retention single intron retention; (H) XIR: Approximate IR single intron retention (fuzzy boundary); (I) MIR: Multi-IR multi-intron retention; (J) XMIR: Approximate MIR multi-intron retention (fuzzy boundary); (K) AE: Alternative exon ends (5′, 3′, or both); (L) XAE: Approximate AE variable 5′ or 3′ end (fuzzy boundary).

### 2.6. Analysis of Differentially Expressed Genes (DEGs)

The level of gene expression for each gene was measured using the FPKM (fragments per kilobase per transcript per million mapped reads), and the FPKM was calculated based on the length of the gene and read counts mapped to the gene. The formula is as follows:

$$\mathrm{FPKM}=\frac{\mathrm{cDNA}\;\mathrm{Fragments}}{\mathrm{Mapped}\;\mathrm{Fragments}(\mathrm{Millions})•\mathrm{Transcript}\;\mathrm{Length}(\mathrm{kb})}$$

Where cDNA Fragments is the number of fragments compared to a transcript; Mapped Fragments (Millions) is the total number of fragments compared to a transcript, in 1 × 10^6^ units; Script Length (kb) is the length of the transcript, in 1 × 10^3^ bases units. The normalized FPKM (FPKM > 1) was used as gene expression level for differential expression analysis by the DESeq software (DESeq2-1.28.1, Bioconductor, Buffalo, NY, USA) (http://www.bioconductor.org/packages/release/bioc/html/DESeq.html; Accessed on: 30 December 2019). A false discovery rate (FDR) was applied to determine the threshold of *p*-value in multiple tests and analyses. The DEGs were evaluated by fold-change ≥ 2 and FDR < 0.01.

### 2.7. Analysis of GO Enrichment and KEGG Pathway Enrichment

GO enrichment analysis of DEGs was implemented using the GOseq R packages (https://bioconductor.org/packages/release/bioc/html/goseq.html; Accessed on: 28 December 2019), and the number of DEGs in each term was calculated. The statistical enrichment of DEGs in the KEGG pathways was tested for further understanding the high-level functions of a biological system by the KOBAS (http://kobas.cbi.pku.edu.cn/kobas3/?t=1; Accessed on: 30 December 2019). Besides, gene functions were annotated with the following databases, including NR (NCBI non-redundant protein sequences, ftp://ftp.ncbi.nih.gov/blast/db/; Accessed on: 30 December 2019); Nt (NCBI non-redundant nucleotide sequences); Pfam (Protein family, http://pfam.xfam.org/; Accessed on: 30 December 2019); KOG/COG (Clusters of Orthologous Groups of proteins, http://www.ncbi.nlm.nih.gov/COG/; http://www.ncbi.nlm.nih.gov/KOG/; Accessed on: 30 December 2019); Swiss-Prot (a manually annotated and reviewed protein sequence database, http://www.uniprot.org/; Accessed on: 30 December 2019); GO (Gene Ontology, http://www.geneontology.org/; Accessed on: 30 December 2019); KO (KEGG Ortholog database).

### 2.8. qPCR Verification

To verify the repeatability and precision in the muscles sampled at different growth stages, 6 DEGs were randomly selected from the transcriptome data for qPCR analysis. The RNA extracted from the same samples with a RIN ≥ 7.3, A260/280 > 1.8, and A260/230 > 2.0, and the first strand of cDNA was synthesized according to the manual of reverse transcription kit (abm, Richmond, Canada). Six pairs of primers and β-actin were designed by the Primer 5.0 software (Table 1), and the stability of β-actin was tested in this system. qPCR was performed using EcoRT48 (OSA, London, UK), and the reaction system comprised 5 μL of 2 × TransStart Tip Green qPCR SuperMix (Transgen, Beijing, China), 0.3 μL of each primer (10 μM), 0.8 μL cDNA (400 ng/μL) and 3.6 μL ddH_2_O, reaching a total volume of 10 μL. The optimal reaction procedure included 95 °C for 30 s, followed by 40 cycles of 95 °C for 5 s, 60 °C for 30 s, then 95 °C for 15 s, 55 °C for 15 s, 95 °C for 15 s. The relative expression levels of DEGs were standardized to the internal reference gene β-actin by 2^−ΔΔCt^ calculation method. Data were presented as mean±SD. The difference was analyzed using one-way analysis of variance (ANOVA) followed by Dunnet’s t-test and Tukey’s test.

## 3. Results

### 3.1. Analysis of RNA-Seq Data

The transcriptomes of breast and leg muscle yielded approximately 316 million (26,340,526 on average) and 318 million (26,550,580 on average) clean reads, respectively. The GC content and Q30 percentages of the breast muscle transcriptomes were 50.72% to 55.32% and 92.56% to 93.71%, respectively, whereas those of the leg muscle transcriptomes were 50.92% to 54.16% and 92.90% to 93.29%, respectively (Table 2).

A total of 1,269,386,566 reads (breast muscle: 632,172,628 with an average of 52,681,052 in each sample, leg muscle: 637,213,938 with an average of 53,101,162 in each sample) were obtained from the transcriptome libraries. The number of mapped reads were 30,008,951 (71.63%) to 48,999,024 (81.85%). While the number of uniquely mapped reads and multiple mapped reads were not less than 28,241,302 (50.04%) and 4,134,800 (9.50%), respectively (Table S3).

### 3.2. Annotation and Classification of SNP/InDel

**Figure 1:**
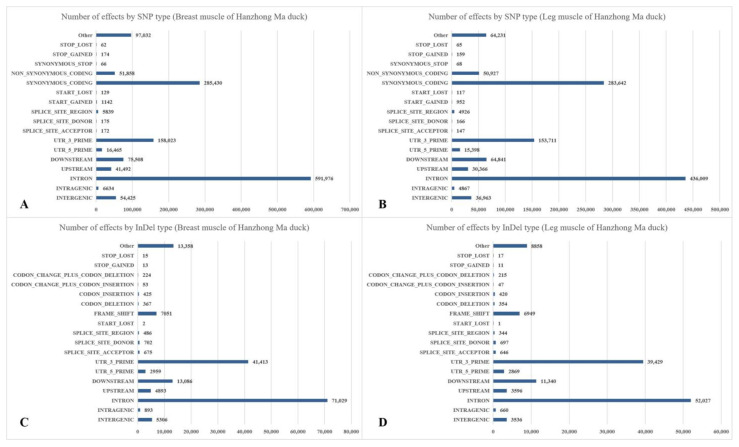
Annotation and classification of SNPs and InDels in Hanzhong Ma ducks. (**A**) SNPs of breast muscle; (**B**) SNPs of leg muscle; (**C**) InDels of breast muscle; (**D**) InDels of leg muscle.

There were 56,327 to 164,499 SNPs in breast muscle, with the genic SNPs ranging from 51,586 to 147,615 and the intergenic SNPs ranging from 4,741 to 16,884, respectively. The total numbers of SNPs in leg muscle were 59,750 to 142,086 (the genic SNPs were 54,384 to 128,232 and the intergenic SNPs were 5,366 to 13,854). Besides, the proportions of transversion-type SNPs in all SNP sites were between 73.51% and 76.06% in breast muscle, and the ratio in leg muscle was between 73.75% and 75.91%. The percentages of the heterozygous SNPs in all SNPs of breast and leg muscle ranged from 35.17% to 41.44% and 36.43% to 41.34%, respectively (Table S4). The most common change was G->A, followed by C->T, A->G, and T->C (Table 3). The annotation results of SNPs and InDels are shown in Figure 1. The first three were “INTRON”, “SYNONYMOUS_CODING”, and “UTR_3_PRIME” in the annotations of SNPs. Besides, the first three were “INTRON”, “UTR_3_PRIME”, and “DOWNSTREAM” in the annotations of InDels.

### 3.3. Prediction of AS events

**Figure 2:**
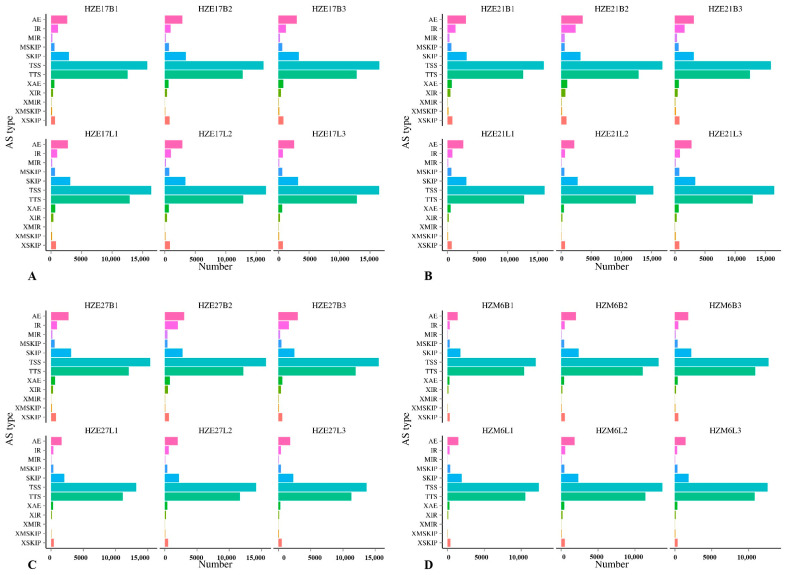
The predicted number of alternative splicing in Hanzhong Ma ducks during different growth stages. (**A**–**D**) represent the predicted number of alternative splicing in Hanzhong Ma ducks on day 17, 21, 27 of incubation, and postnatal 6-month-old, respectively.

In skeletal muscle of Hanzhong Ma ducks at the embryonic day 17, 21, 27, and postnatal 6-months-old, the predicted number of AS were mainly concentrated in alternative 5′ first exon (transcription start site)-the first exon splicing (TSS) and alternative 3′ last exon (transcription terminal site)-the last exon splicing (TTS), indicating that TSS and TTS were the most common AS events in Hanzhong Ma ducks (Figure 2).

### 3.4. Gene Functional Annotation and Classification

Based on the sequence of *Anas platyrhynchos* genome, mapped reads were spliced by the StringTie software (https://ccb.jhu.edu/software/stringtie/index.shtml; Accessed on: 30 December 2019). In order to complement and improve the previous genome annotated information, these reads were compared with the *Anas platyrhynchos* genome annotated information to discover uncommented transcription regions and the new genes. Most notably, there were 292 to 2801 annotated DEGs in breast muscle and 304 to 1950 annotated DEGs in leg muscle from different databases (Table 4).

### 3.5. Analysis of Differential Expressed Genes

**Figure 3:**
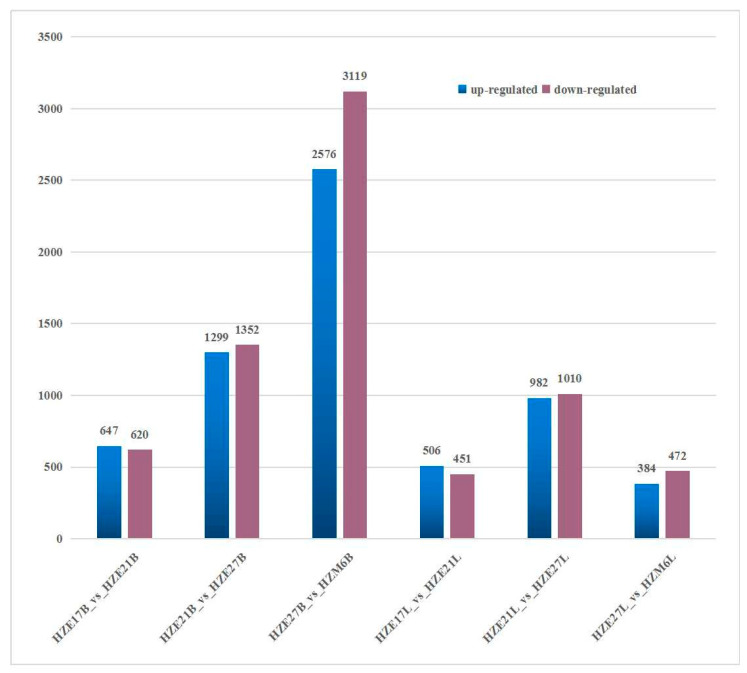
Number of DEGs during skeletal muscle development in Hanzhong Ma ducks.

**Figure 4:**
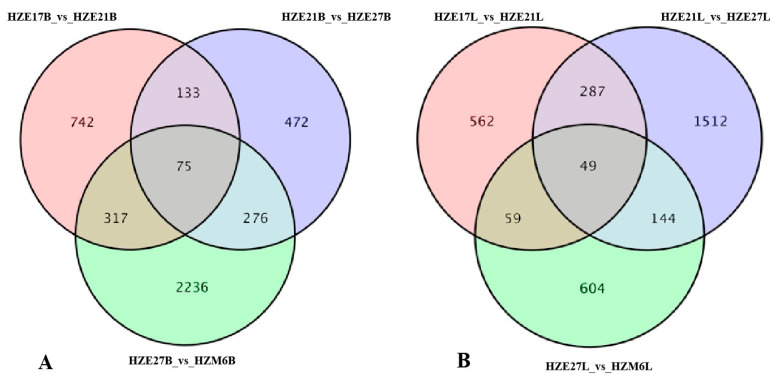
Venn diagram of DEGs among the three comparison groups. (**A**) The co-expressed DEGs in breast muscle of Hanzhong Ma ducks; (**B**) The co-expressed DEGs in leg muscle of Hanzhong Ma ducks.

DEGs were identified by taking fold-change ≥ 2 and FDR < 0.01 as the cutoff. In the breast muscle of Hanzhong Ma ducks, a total of 1267 DEGs were detected, with 647 up-regulated genes and 620 down-regulated genes in HZE17B_vs_HZE21B. There were 2651 DEGs significantly differentially expressed in HZE21B_vs_HZE27B, which include 1299 up-regulated genes and 1352 down-regulated genes. Moreover, 5695 DEGs were identified in HZE27B_vs_HZM6B, among which 2576 were up-regulated genes and 3119 were down-regulated genes. In leg muscle, a total of 957 DEGs were found in HZE17L_vs_HZE21L, of which 506 were up-regulated genes and 451 were down-regulated genes. A total of 1992 DEGs were detected in HZE21L_vs_HZE27L, and the up-regulated genes were 982 and the down-regulated genes were 1010. In addition, 856 DEGs were discovered in HZE27L_vs_ HZM6L, and 384 DEGs were up-regulated genes and 472 DEGs were down-regulated genes (Figure 3). Notably, a total of 75 DEGs in breast muscle and 49 DEGs in leg muscle were co-expressed at all developmental points of comparison, respectively (Figure 4).

### 3.6. GO Annotation and KEGG Pathway Analysis

**Figure 5:**
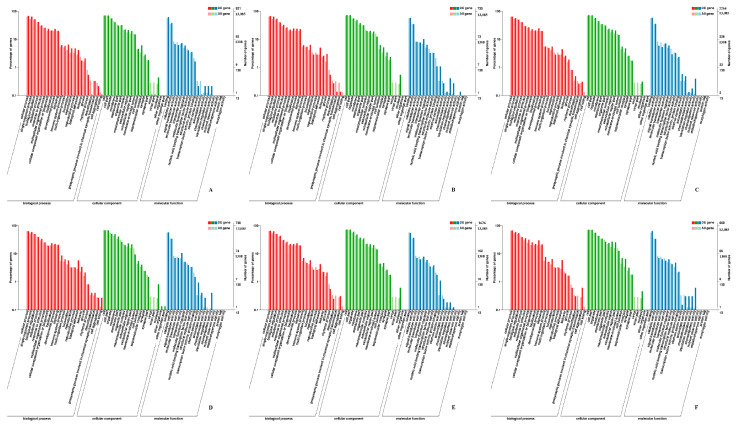
GO enrichment analysis of DEGs in Hanzhong Ma ducks. (**A**) HZE17B_vs_HZE21B; (**B**) HZE21B_vs_HZE27B; (**C**) HZE27B_vs_HZM6B; (**D**) HZE17L_vs_HZE21L; (**E**) HZE21L_vs_HZE27L; (**F**) HZE27L_vs_HZM6L. Note: The abscissa was GO terms, the ordinate on the left was the percentage of genes in all genes annotated with GO, right was the number of genes.

GO and KEGG analysis was performed to further understand the biological functions of the genes within the significant gene expression profiles. The DEGs were categorized into three main GO categories: biological process, cellular component, and molecular function (Figure 5). Results show that “myosin complex”, “muscle-tendon junction” and “myofibril” were significantly enriched in the cellular component category. As for the molecular function category, most DEGs were assigned to “extracellular matrix structural constituent”, “microtubule motor activity”, and “muscle alpha-actinin binding”. In the biological process, the DEGs were significantly enriched in “negative regulation of skeletal muscle tissue development”, “embryonic skeletal system morphogenesis”, and “regulation of skeletal muscle contraction” (Table S5). These RNA-Seq results reaffirmed the differential expression of several genes between skeletal muscles at different growth stages, such as *CREBL*2, *RHEB*, *GDF*6, *SHISA*2, and *MYLK*2. In addition, the genes *ACTN*3, *RYR*3, and *STMN*1, which play crucial roles in muscle development, were observed to be differentially expressed. The identified DEGs might function in transcriptional regulation of skeletal muscle development at different growth stages.

**Figure 6:**
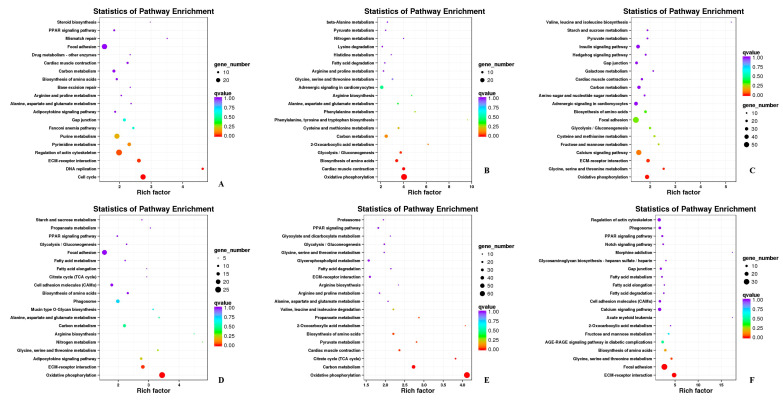
Annotation of DEGs in KEGG. (**A**) HZE17B_vs_HZE21B; (**B**) HZE21B_vs_HZE27B; (**C**) HZE27B_vs_HZM6B; (**D**) HZE17L_vs_PHZE21L; (**E**) HZE21L_vs_HZE27L; (**F**) HZE27L_vs_HZM6L. Note: In the figure, each circle represented a KEGG pathway, the name of which was shown on the left legend. Abscissa was enrichment factors, showing the proportion of (a) to (b), (a) was the ratio of differentially expressed genes in the pathway with all DEGs in all pathways; (b) was the ratio of genes in the pathway with all genes in all pathways. The bigger the Rich factor is, the more significant the pathway is. The color of the circle represented the q value which is the adjusted *p*-value by multiple hypothesis testing. Thus, the smaller the q value is, the more significant the pathway is; the circle size represented the number of differentially expressed genes annotated with the pathway, the bigger circle size is, the higher the number of genes.

The KEGG pathways of DEGs were shown in Table S6. The enriched pathways included “regulation of actin cytoskeleton”, “oxidative phosphorylation”, “carbon metabolism”, “calcium signaling pathway”, “focal adhesion”, “ECM-receptor interaction”, and “MAPK signaling pathway”, in which “regulation of actin cytoskeleton”, “oxidative phosphorylation”, and “focal adhesion” were significantly enriched (Figure 6).

### 3.7. Validation of RNA-Seq Results

**Figure 7:**
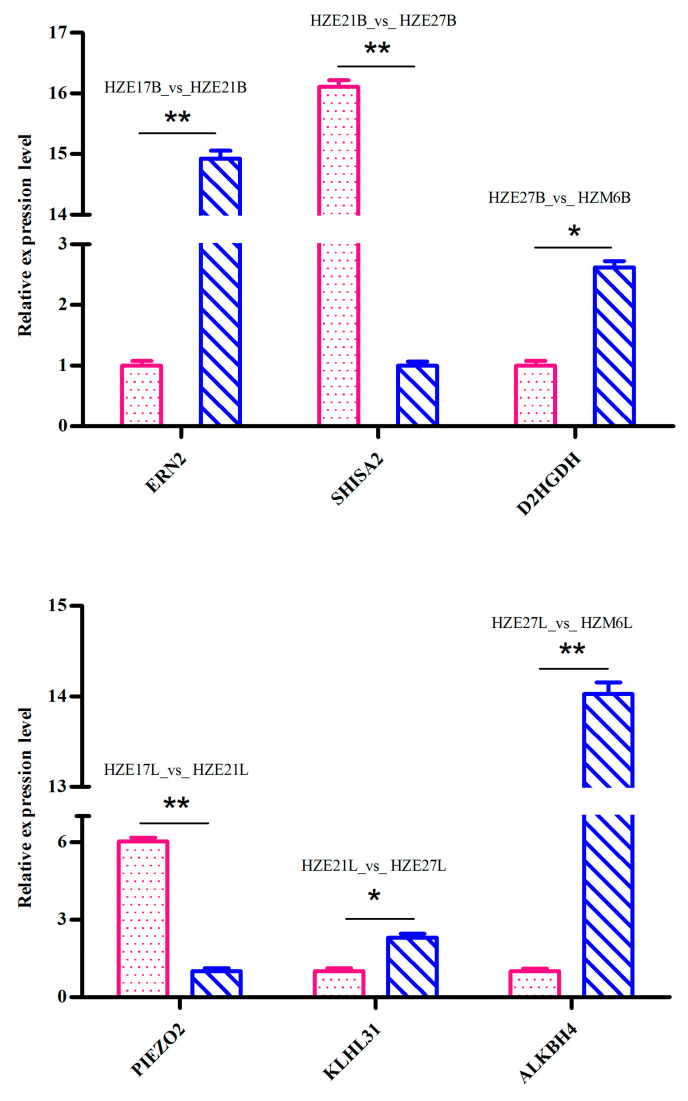
qPCR verification of DEGs. “*” was considered significant difference (*p* < 0.05); “**” was considered extremely significant difference (*p* < 0.01).

To validate the reliability of results from RNA-Seq, 6 DEGs (*ERN*2, *D2HGDH*, *KLHL*31, *ALKBH*4, *SHISA*2, and *PIEZO*2) were randomly selected to further examine using qPCR. All the selected DEGs showed concordant expression patterns between the RNA-Seq and qPCR results (Figure 7).

## 4. Discussion

Because meat yield directly determines the level of economic income, there has recently been great interest in achieving higher growth performance and better meat quality in livestock and poultry [19,20]. Muscle development is strictly regulated by related genes, transcriptional regulatory factors, or non-coding RNAs (lncRNA or circRNA) [21,22,23]. In this study, a transcriptome level analysis of mRNAs was performed in breast and leg muscles of female Hanzhong Ma ducks at four distinct stages, and a comprehensive understanding of the dynamic changes was obtained from the transcriptome during muscle development. The reason why we chose female ducks was that they account for the majority of duck farms, and the same sex can avoid errors in sequencing data. Transcriptomic sequencing data from breast and leg muscles of Hanzhong Ma ducks at all time points received 632,172,628 and 637,213,938 reads, respectively. The percentages of aligned reads to the *Anas platyrhynchos* genome were high, over 71.63% and 77.36%, respectively. This suggests that the sequencing data have high coverage and the cDNA libraries were created successfully. In gene functional annotation and classification, there were 292 to 2801 annotated DEGs in the breast muscle and 304 to 1950 annotated DEGs in the leg muscle from different databases, which can greatly supplement and improve the annotation information of the duck genome. The expression profiles of the genes chosen for qPCR verification were entirely consistent with the transcriptome results, indicating that the transcriptome data were valid.

### 4.1. Annotation and Classification of SNP/InDel in Skeletal Muscle Developmental Process

SNP is a kind of efficient genetic marker based upon the variability at the nucleotide level and it has been widely used in genetic studies and breeding applications in animal species [24,25]. SNPs are more abundant in organisms, which have greater stability over generations and are more accurately genotyped. InDel mutation is defined by the addition or loss of one or more nucleotides of a DNA sequence throughout the genome and it contains valuable phylogenetic information. InDels in the coding regions of a gene can either cause frameshifts or amino acid insertions/deletions, which may affect protein function [26]. Therefore, it is meaningful to study the roles of SNPs and InDels in duck skeletal muscle development.

Based on the alignment of the reads to the *Anas platyrhynchos* genome, the potential SNP sites and the InDels were identified by using the GATK software. A total of 56,327 to 164,499 SNPs in breast muscle and 59,750 to 142,086 SNPs in leg muscle were detected. The first four changes were G->A, followed by C->T, A->G, and T->C. The annotation results of SNP were mainly “INTRON”, “SYNONYMOUS_CODING”, and “UTR_3_PRIME”. It is helpful for us to understand the most common base changes and the types of SNP annotations in duck skeletal muscle, which can be used as important molecular markers in the genetic breeding of Hanzhong Ma duck. Besides, the types of InDel annotations in skeletal muscle of Hanzhong Ma duck were “INTRON”, “UTR_3_PRIME”, and “DOWNSTREAM”, which may play an important role in the evaluation of duck economic traits. Through continued research, the findings of these SNPs and InDels may reveal genomic markers controlling genetic variation in economically important duck muscle phenotypes by improving our knowledge of the underlying trait biology.

### 4.2. AS Events in Skeletal Muscle Developmental Process

AS is a tightly regulated biological process and it is the main source of transcriptome and proteome diversity, which largely contributes to the complexity of eukaryotes [27]. AS of eukaryotic pre-mRNA is an important mechanism for regulating tissue- or development-specific gene expression [28], and it greatly expands the coding capacities of genetic information. By using different splicing sites, two or more mRNAs are generated from the same pre-mRNA, that is, many varied proteins are produced from a single gene. The resulting mRNAs have distinct regulatory functions in the cell, such as localization, stability, and translational efficiency [29]. Therefore, AS plays an important role in the regulatory mechanism and functional properties of eukaryotic organisms [30]. Fiszbein A et al. found that AS played a role in myogenic differentiation [31]. At present, there are few functional studies about the AS events in duck at different growth stages. In this study, the TSS and TTS of the AS events were the most (>10,000) in skeletal muscle at embryonic day 17, 21, 27, and at postnatal 6-month-old, suggesting that they are the most common AS events in skeletal muscle of Hanzhong Ma duck. The identification of AS events will contribute to a better understanding of the regulatory mechanisms during the duck skeletal muscle myogenesis.

### 4.3. DEGs Analyzed at All Time Points

Differential gene expression is considered to be the main cause of genetic variation in animal muscle development, indicating that the regulation mechanism of muscle development may have changed. Xue Q et al. identified the candidate genes involved in the muscle growth of chicken and found that *MYOD*1, *GH*, *IGF2BP*2, *IGFBP*3, *SMYD*1, *CEBPB*, *FGF*2, and *IGFBP*5 were well known to be related to chicken growth [7]. Ayuso M et al. investigated the gene expression and transcriptional regulation of pigs at two developmental stages, several genes, such as *SIM*1, *PVALB*, MEFs, *TCF7L*2, *FOXO*1, *PVALB*, *KLF*1, or *IRF*2 were identified, which were involved in muscle tissues development [32]. According to the DEGs of Hanzhong duck skeletal muscle at different growth stages, several genes that may affect the development of skeletal muscle were identified, such as *CREBL*2, *RHEB*, *GDF*6, *SHISA*2, *MYLK*2, *ACTN*3, *RYR*3, and *STMN*1. In addition, some studies have shown that these regulatory transcription factors interacted with each other in regulating muscle development.

The cAMP-response element-binding protein (CREB), a direct downstream target for AMPK [33], participates in metabolic regulation [34]. Many growth factors and inflammatory signals induce the activation and expression of *CREB* and then mediate the transcription of various genes containing cAMP response elements [35]. Pugazhenthi S et al. indicated that *IGF*I induced anti-apoptotic protein Bcl-2 promoter activity through *CREB*. Similarly, CREB-like 2 (CREBL2) is also a regulator of cellular metabolism [36]. Tiebe M et al. found that *CREBL*2 regulated cell metabolism of C2C12 myoblasts [37]. Ras homolog enriched in the brain (RHEB), a monomeric protein with a molecular weight of about 21 kDa, is broadly expressed in human and animal tissues [38]. It is well known that rapamycin complex 1 (mTORC1) is a major regulator of cell growth and metabolism, and the small G protein *RHEB* activates mTORC1 in response to growth factor signals [39,40]. Besides, the Rheb-mTOR/Raptor pathway negatively regulates myogenic differentiation by inhibiting the IRS1-PI3K-AKT signaling pathway [41]. Under the induction of amino acids and insulin, the formation of the RHEB-mTOR complex in the skeletal muscle of newborn piglets may be related to the activation of mTORC1 that regulates skeletal muscle protein synthesis [42]. MacLea KS et al. suggested that *RHEB* regulated the development of skeletal muscle in the blackback land crab [43]. Growth/differentiation factors 6 (GDF6), also known as bone morphogenetic protein (BMP13) and cartilage-derived morphogenetic protein (CDMP-2), forms part of the transforming growth factor-β superfamily and is highly expressed during embryogenesis [44]. *GDF*6 is highly conserved in vertebrates and has been shown to play a key role in limb joint formation and chondrogenesis. It is expressed in many mesenchymal derivatives, such as the tendon and cartilage, but is less expressed in the intestine, skeletal muscle, and placenta. Mikic B et al. demonstrated that *GDF*6 without mutation was associated with a significant decrease of collagen content in the tail tendon of 4-week-old male mice [45]. Shisa family member 2 (SHISA2) antagonizes FGF and Wnt signaling [46]. *SHISA*2 promotes the maturation and transition of somitic precursors in Xenopus laevis embryos by individually inhibiting Wnt and FGF signal transduction [47]. Similarly, *SHISA*2 affects chicken embryo development by regulating FGF and Wnt signaling [48], and its expression is regulated by Notch signal [49]. The deletion of *SHISA*2 inhibits the fusion of myoblasts without affecting the proliferation, and overexpression of *SHISA*2 inhibits its proliferation and promoted premature fusion.

Myosin light chain kinase 2 (MYLK2) encodes a calcium/calmodulin-dependent serine/threonine kinase (myosin light chain kinase, MLCK), which is expressed in skeletal muscle and cardiac muscle [50] and activates the actin contraction with myosin. With the increase of local Ca^2+^ concentrations, the sarcoplasmic reticulum releases large amounts of Ca^2+^, which binds to troponin C followed by myosin-actin cross-bridge formation. In this process, MLCK enhances the peak tension of skeletal muscle, as well as the force and rate of cross-bridge recruitment of cardiomyocytes [51,52,53]. Zhang XM et al. revealed that *MYLK*2 played a vital role in regulating distinct early porcine embryonic myogenic processes between Wuzhishan and Landrace pigs [54]. ACTNs (α-actinins) are widely expressed cytoskeleton proteins that cross-link actin filaments at the adherens junctions in epithelial and focal adhesions at the leading edge of migrating cells [55,56], and play a key role in the maintenance and regulation of the cytoskeleton. In mammalian cells, there are four kinds of ACTNs, which are the components of all three kinds of stress fibers (ventral stress fibers, dorsal stress fibers, and transverse arcs) [57,58]. *ACTN*3 is a prominent actin filament associated protein that is expressed in skeletal muscle [59], and its function is to maintain the microfilament spacing at the Z-disc. Mice lacking *ACTN*3 activity show a shift to aerobic metabolism, resulting in efficient muscle function and increased endurance [60]. Holterhoff CK et al. suggested that the variation of *ACTN*3 expression may promote the physiological diversity of vertebrate muscle function [61]. RyR (ryanodine receptor), a Ca^2+^ release channel in the sarcoplasmic reticulum in vertebrate skeletal muscle, plays an important role in excitation-contraction coupling [62]. *RYR*3 is expressed at low levels in the brain, smooth muscle, and slow-twitch skeletal muscle [63]. The expression of *RYR*3 is related to the augmented spontaneous Ca^2+^ activity in muscle fibers and cultured myotubes, and it is also associated with increased frequency and size of Ca^2+^ sparks [64,65]. Perni S et al. found that *RY*R3 may play a preferred role in physiological processes of CICR (Ca^2+^-induced Ca^2+^ release) in skeletal muscle and some other tissues [66]. In avian skeletal muscle, *RYR*1 and *RYR*3 are expressed simultaneously [67]. STMN1 (Stathmin1), a cytosolic phosphoprotein, is involved in regulating the microtubule dynamics in response to the cell’s need [68] and plays an important role in mitotic spindle formation and cell mitosis [69]. The expression of *STMN*1 is strongly regulated during tissue development and maturation, and it is considered to be a general relay integrating diverse intracellular signaling pathways [70]. *STMN*1 has been shown to significantly reduce the SMA (spinal muscular atrophy) phenotype independent of restoring SMN protein [71].

All these studies have identified that the DEGs have important functions in skeletal muscle development. Therefore, the differential expression of genes that were in the breast and leg muscles of Hanzhong Ma ducks, may have an important regulatory effect on the mechanism of skeletal muscle development.

### 4.4. Analysis of GO and KEGG Pathway

The KEGG pathways play crucial roles in muscle growth and development in animals. Wu PF et al. carried out a transcriptome study on the breast muscles of Jinghai yellow chickens and they found that extracellular matrix (ECM)-receptor interaction, the mitogen-activated protein kinase (MAPK) signaling pathway, and focal adhesion were the most enriched for the DEGs in KEGG pathway enrichment [72]. Zhang ZR et al. also found that the ECM-receptor interaction, MAPK signaling pathway, and focal adhesion, were the most enriched DEGs in breast muscle of chickens [15]. Zhao YQ et al. found that focal adhesion, protein digestion and absorption, GABAergic synapse, axon guidance, ECM-receptor interaction, MAPK signaling pathway, arginine, and proline metabolism were closely related to skeletal muscle of Tongcheng pigs, while oxidative phosphorylation, Huntington’s disease, ribosome biogenesis in eukaryotes, metabolic pathways, Alzheimer’s disease, glycolysis/gluconeogenesis, and proteasome were found to be more associated with the DEGs in skeletal muscle of Yorkshire pigs at developmental stages [73]. The biological processes categories in this study, including “negative regulation of skeletal muscle tissue development”, “embryonic skeletal system morphogenesis”, and “regulation of skeletal muscle contraction”, were significantly regulated by the DEGs, indicating that these DEGs played a key role in regulating duck skeletal muscle development at different stages. KEGG pathways, including regulation of actin cytoskeleton, oxidative phosphorylation, and focal adhesion were confirmed to be involved in the skeletal muscle development of the Hanzhong Ma duck.

Focal adhesions (FAs) are dynamic macromolecular structures based on large integrin, which connect the extracellular matrix (ECM) with the intracellular actin cytoskeleton. The FA components include the linkage between the integrin receptor and the actin cytoskeleton, which determine the dynamics of FAs (the formation, maturation, and disassembly of FAs) and the organization of the cytoskeleton [74]. FAs provide traction and transmit signals that drive cell migration by expanding and altering its composition, which is crucial for a variety of biological processes, including development, wound healing, and cancer metastasis. FA-related signaling networks dynamically regulate the strength of the linkage between integrin and actin and control the organization of the actin cytoskeleton [75,76]. Focal adhesion kinase (FAK) transmits two-way signals at FAs between the ECM and the intracellular milieu, and the FAK complex plays a signal role in the triggering of adaptive changes in the fiber. Activation of FAK can initiate intracellular signal transduction cascade, including those involved in the mitogen-activated protein kinase (MAPK) effector cascades and cytoskeleton remodeling, which in turn regulate cell migration, growth, and differentiation [77]. Besides, FAK is highly overexpressed in hypertrophied skeletal muscle [78], which is activated by integrin-mediated cell adhesion to the ECM and stimulates the activity of a variety of intracellular signaling pathways, such as paxillin and phosphatidylinositol-3 kinase (PI3K) pathways. Quach NL et al. found that FAK signaling was essential for both costamerogenesis and myofibrillogenesis in differentiated skeletal muscle cells in vitro [79]. Wang D et al. showed that FAK and paxillin promoted migration and adhesion of swine skeletal muscle satellite cells to fibronectin [80]. Therefore, FAs may play a key role in duck muscle development in this study.

Cell motility plays a central role in many biological processes, such as embryonic development, tissue repair, immune response, and cancer metastasis. Motility requires precise integration and regulation of various cellular processes, including dynamic cytoskeleton remodeling [81,82]. The cytoskeleton is a dynamic filamentous system containing actin filaments and microtubules, and it is an integral part of skeletal muscle structure [83]. The contraction of skeletal muscle depends on the release of Ca^2+^ [84]. Johnson BD et al. found that hormones and neurotransmitters may interact with the cytoskeleton in a key way to regulate Ca^2+^ channel activity through the PKA signal transduction pathway [85]. The actin cytoskeleton forms a very dynamic structural network, which is constantly remodeled in eukaryotic cells to control and coordinate a variety of cellular processes, including the establishment and maintenance of cell polarity, polarized cell migration, cell adhesion, cytokinesis, and intracellular transport [86,87]. In this study, the function of the actin cytoskeleton may be essential in the development of duck skeletal muscle.

Oxidative phosphorylation (OXPHOS) is the main process responsible for ATP production in most animal cells. Skeletal muscle is a kind of tissue with high energy requirement, and OXPHOS plays an important role in skeletal muscle energy homeostasis under various physiological conditions [88,89,90]. Skeletal myoblasts specifically change from a highly glycolytic state to mainly dependent on OXPHOS upon differentiation [91]. In skeletal muscle, slow-twitch fibers (type Ⅰ) and fast-twitch fibers (type Ⅱa) are rich in mitochondria, and their ATP supply mainly depends on OXPHOS, while fast-twitch fibers (type Ⅱb) lack mitochondria and are mainly produced by effective glycolytic ATP [92,93]. Ca^2+^ is believed to regulate mitochondrial OXPHOS, which helps to maintain cell energy homeostasis [94]. Besides, Vinnakota KC et al. found that Ca^2+^ can regulate OXPHOS of skeletal muscle mitochondria [95]. Therefore, during the embryonic stages, the development of duck skeletal muscle requires a large amount of energy supply, and OXPHOS may be an important energy supply pathway.

## 5. Conclusions

In this study, transcriptome sequencing of Hanzhong Ma duck skeletal muscle at different growth stages was carried out. A large number of SNPs, InDels, and AS events were detected across all the stages. Several genes that may be important candidate genes involved in skeletal muscle development were screened and discussed, such as *CREBL*2, *RHEB*, *GDF*6, *SHISA*2, *MYLK*2, *ACTN*3, *RYR*3, and *STMN*1. Besides, the key regulatory pathways, namely, regulation of actin cytoskeleton, oxidative phosphorylation, and focal adhesion, played a crucial regulatory role in skeletal muscle development. This study is helpful for understanding the genetic architecture of the Hanzhong Ma duck transcriptome and provides a useful resource and markers for functional genomics research in the future. At the same time, the results may help us understand the developmental molecular process of skeletal muscle, which is an economically important carcass trait for duck production.

## Figures and Tables

**Table 1:** Primers sequence used in this work for qPCR validation and sex-determination.

Groups	Primer Name	Primer Sequence (5′-3′)	Size	Regulated
	gCHD	F: TGCAGAAGCAATATTACAAGT	Male: 467 bp	
		R: AATTCATTATCATCTGGTGG	Female: 467 bp, 326 bp	
HZE17B_vs_HZE21B	*ERN*2	F:GCTACCTCACCTTCCACTCG	144 bp	UP
		R:CCAGTGAGGTCAAGGCGTAG		
HZE21B_vs_ HZE27B	*SHISA*2	F:AACTCTGTCTCTTGGCGGAC	140 bp	DOWN
		R:GAAGTCGCAGCACAACCTTC		
HZE27B_vs_ HZM6B	*D2HGDH*	F:CTACGGCCACTTGGGAGATG	138 bp	UP
		R:CCATGCTCGGCACTGATACT		
HZE17L_vs_ HZE21L	*PIEZO*2	F:GAGGGAGTTCGTGAGTGGTG	153 bp	DOWN
		R:CGATGCGTACAGTCCCATGA		
HZE21L_vs_ HZE27L	*KLHL*31	F:AACCAGTGCGTGACAGTGAT	171 bp	UP
		R:GCTGAAGTGGGTACGCTTCT		
HZE27L_vs_ HZM6L	*ALKBH*4	F:CTTGCTCTGTGCTAGGTGGT	156 bp	UP
		R:TGGAGAGCACGGTGTTTGAG		
	*β-actin*	F: CCCTGTATGCCTCTGGTCG	194 bp	
		R: CTCGGCTGTGGTGGTGAAG		

**Table 2:** RNA-Seq data from breast and leg muscle of Hanzhong Ma ducks.

Samples	Clean Reads	Clean Bases	GC Content	Q30 Value
HZE17B1	21,762,267	6,501,302,702	51.05%	93.11%
HZE17B2	27,394,948	8,181,272,166	50.72%	93.71%
HZE17B3	29,162,348	8,705,985,402	51.30%	93.06%
HZE17L1	27,479,839	8,207,191,364	51.34%	92.90%
HZE17L2	27,736,375	8,286,040,228	50.92%	93.27%
HZE17L3	24,349,210	7,267,672,312	51.27%	93.04%
HZE21B1	26,420,707	7,891,652,864	51.40%	92.56%
HZE21B2	28,097,657	8,385,363,516	51.13%	93.00%
HZE21B3	27,589,171	8,240,063,270	51.19%	92.73%
HZE21L1	26,743,965	7,984,802,282	51.25%	93.27%
HZE21L2	22,304,168	6,655,180,216	51.39%	93.03%
HZE21L3	29,933,693	8,920,698,448	51.09%	92.93%
HZE27B1	30,600,812	9,149,184,640	52.14%	92.99%
HZE27B2	25,569,769	7,639,778,632	51.34%	92.58%
HZE27B3	27,794,014	8,301,392,200	51.66%	92.72%
HZE27L1	26,774,058	7,994,219,756	51.77%	93.02%
HZE27L2	27,147,241	8,098,721,002	52.00%	93.29%
HZE27L3	27,135,496	8,108,815,318	52.42%	92.97%
HZM6B1	20,948,194	6,261,607,884	55.32%	93.28%
HZM6B2	28,678,868	8,557,762,996	54.45%	93.16%
HZM6B3	22,067,559	6,594,491,658	53.08%	93.25%
HZM6L1	25,190,674	7,526,783,110	54.01%	93.18%
HZM6L2	27,248,765	8,136,824,248	52.27%	93.05%
HZM6L3	26,563,485	7,931,120,332	54.16%	92.96%

Note: Clean reads: Paired-end numbers of Clean Data; HZE17B: Breast muscle of Hanzhong Ma duck on the day 17 of the incubation period; HZE17L: Leg muscle of Hanzhong Ma duck on the day 17 of the incubation period; The same below.

**Table 3:** Single nucleotide polymorphisms (SNP) mutation type from breast and leg muscle of Hanzhong Ma ducks.

Sample	A->G	G->A	C->T	T->C	A->C	C->A	A->T	T->A	C->G	G->C	G->T	T->G
HZE17B1	22,365	23,409	23,406	22,291	3902	4103	3284	3258	3810	3774	3952	3955
HZE17B2	23,579	24,692	24,688	23,381	4229	4342	3393	3473	4009	3980	4124	4263
HZE17B3	25,022	26,021	26,262	25,165	4530	4626	3643	3755	4257	4312	4451	4568
HZE17L1	23,222	24,485	24,837	23,694	4132	4398	3413	3404	4115	3934	4160	4200
HZE17L2	25,603	26,918	26,759	25,514	4713	4804	3770	3793	4454	4419	4550	4692
HZE17L3	20,922	21,871	22,038	20,657	3651	3664	2953	3046	3545	3503	3710	3644
HZE21B1	25,318	26,301	26,223	24,981	4599	4627	3768	3792	4332	4305	4558	4565
HZE21B2	29,584	30,912	30,895	29,527	5437	5481	4561	4514	5129	5147	5465	5602
HZE21B3	23,938	24,986	24,785	23,657	4362	4392	3559	3586	4089	4138	4289	4440
HZE21L1	20,592	21,845	21,750	20,480	3677	3779	2921	3052	3547	3462	3659	3633
HZE21L2	17,187	18,200	18,172	17,303	2909	2978	2377	2385	2843	2743	3021	2993
HZE21L3	20,918	22,245	22,658	21,283	3654	3811	3070	3130	3603	3630	3754	3836
HZE27B1	21,716	22,786	22,936	21,570	3837	3885	3126	3095	3670	3597	3848	3847
HZE27B2	24,859	25,867	25,853	24,937	4571	4601	3837	3772	4178	4276	4596	4602
HZE27B3	18,307	19,263	19,446	18,163	3217	3223	2591	2650	3056	2965	3231	3255
HZE27L1	12,356	13,325	13,428	12,510	2031	2029	1657	1693	1970	1914	2001	2034
HZE27L2	15,727	16,779	16,715	15,626	2644	2676	2125	2204	2644	2531	2735	2719
HZE27L3	13,577	14,624	14,728	13,583	2162	2250	1821	1772	2207	2149	2232	2187
HZM6B1	10,199	11,011	11,094	10,541	1627	1665	1318	1380	1582	1547	1667	1693
HZM6B2	13,446	14,322	14,200	13,523	2222	2248	1862	1903	2166	2167	2265	2254
HZM6B3	12,499	13,708	13,539	12,882	2129	2164	1753	1732	2080	1987	2047	2112
HZM6L1	10,989	11,662	11,732	10,975	1779	1794	1461	1464	1704	1660	1741	1779
HZM6L2	15,108	16,062	15,937	14,998	2510	2595	2073	2169	2522	2452	2583	2583
HZM6L3	11,394	12,207	12,301	11,403	1837	1860	1521	1511	1808	1783	1817	1815

**Table 4:** Annotated number of DEGs in breast muscle and leg muscle of Hanzhong Ma ducks.

DEG Set	Total	COG	GO	KEGG	KOG	NR	Pfam	Swiss-Prot	eggNOG
HZE17B_vs_HZE21B	1190	381	922	787	813	1186	1044	863	1123
HZE21B_vs_HZE27B	919	292	736	613	637	916	847	628	881
HZE27B_vs_HZM6B	2801	965	2266	1889	2041	2784	2581	1992	2728
HZE17L_vs_HZE21L	917	304	741	619	615	912	853	678	894
HZE21L_vs_HZE27L	1950	710	1627	1398	1430	1939	1843	1407	1915
HZE27L_vs_ HZM6L	825	277	660	579	589	820	777	627	808

## Data Availability

The datasets generated for this study can be found in the NCBI SRA. The Submission of Hanzhong Ma duck: SUB8201145 and SUB8202437, Bioproject #PRJNA665331 and Biosamples #SAMN16251816-SAMN16251827 (Breast muscle); Bioproject #PRJNA665334 and Biosamples #SAMN16251828-SAMN16251839 (Leg muscle).

## Supplementary Materials

The following are available online at https://www.mdpi.com/2218-273X/11/2/315/s1, Table S1. The feed composition of duck; Table S2. The concentrations and RINs of sample RNA; Table S3. Statistics of sequence comparison between sample sequencing data and *Anas platyrhynchos* genome; Table S4. SNPs from breast and leg muscle of Hanzhong Ma duck; Table S5. The most enriched GO terms related to muscle development; Table S6. Top 5 in KEGG enrichment.
