# Supplementary material for: Skeletal Muscle Transcriptome Analysis of Hanzhong Ma Duck at Different Growth Stages Using RNA-Seq
Source: Biomolecules. 2021 Feb 19;11(2):315. doi: 10.3390/biom11020315 (PMC7927120; doi:10.3390/biom11020315)
Supplement: Supplementary file 1 [file biomolecules-11-00315-s001.zip › biomolecules-1104004-supplementary/Supplementary Materials/Table S1.docx]

**Table S1.** The feed composition of duck.

| **Ingredient** | **Content (%)** | **Nutrient** | **Content (%)** |
| --- | --- | --- | --- |
| Corn | 56.00 | Crude protein | 15.700 |
| Soybean meal | 23.80 | Calcium | 0.900 |
| Corn gluten meal | 10.00 | Total phosphorus | 0.680 |
| Limestone | 7.00 | Available phosphorus | 0.450 |
| CaHPO_4_ | 1.50 | Salt | 0.370 |
| Premix | 1.00 | Lysine | 0.760 |
| NaCl | 0.30 | Methionine | 0.387 |
| Lys·HCl | 0.30 | Methionine + Cystine | 0.654 |
| *DL*-Met | 0.10 | Isoleucine | 0.534 |
| Total | 100.00 | Threonine | 0.579 |
|  |  | Tryptophan | 0.194 |
|  |  | Crude fiber | 4.100 |
|  |  | Crude fat | 3.400 |
|  |  | Crude ash | 5.200 |
|  |  | Avian metabolizable energy | 2875 Mcal·kg^−1^ |

**Note:** Supplied per kilogram of total diet: Cu (CuSO_4_•5H_2_O), 8 mg; Fe (FeSO_4_•7H_2_O), 52 mg; Zn (ZnO), 60 mg; Mn (MnSO_4_•H_2_O), 80 mg; Se (NaSeO_3_), 0.3 mg; I (KI), 0.2 mg; choline chloride, 1,000 mg; vitamin A (retinyl acetate), 10,000 IU; vitamin D3 (Cholcalciferol), 3,000 IU; vitamin E (DL-α-tocopheryl acetate), 20 IU; vitamin K3 (menadione sodium bisulfate), 2 mg; thiamin (thiamin mononitrate), 2 mg; riboflavin, 10 mg; pyridoxine hydrochloride, 4 mg; cobalamin, 0.02 mg; calcium-D-pantothenate, 20 mg; nicotinic acid, 50 mg; folic acid, 1 mg; biotin, 0.2 mg.
