# Supplementary material for: Skeletal Muscle Transcriptome Analysis of Hanzhong Ma Duck at Different Growth Stages Using RNA-Seq
Source: Biomolecules. 2021 Feb 19;11(2):315. doi: 10.3390/biom11020315 (PMC7927120; doi:10.3390/biom11020315)
Supplement: Supplementary file 1 [file biomolecules-11-00315-s001.zip › biomolecules-1104004-supplementary/Supplementary Materials/Table S2.docx]

**Table S2.** The concentration and RIN value of sample RNA

| Breast Muscle | Concentration (ng/μL) | RIN value | Leg Muscle | Concentration (ng/μL) | RIN value |
| --- | --- | --- | --- | --- | --- |
| HZE17B1 | 196.1 | 9.1 | HZE17L1 | 2428.5 | 9.1 |
| HZE17B2 | 1131.8 | 9.4 | HZE17L2 | 703.4 | 9.2 |
| HZE17B3 | 1298.6 | 9.3 | HZE17L3 | 1106.9 | 9.1 |
| HZE21B1 | 508.8 | 9.0 | HZE21L1 | 256.0 | 9.0 |
| HZE21B2 | 427.0 | 8.8 | HZE21L2 | 481.6 | 9.0 |
| HZE21B3 | 516.4 | 8.6 | HZE21L3 | 884.4 | 8.5 |
| HZE27B1 | 474.2 | 8.2 | HZE27L1 | 514.6 | 8.7 |
| HZE27B2 | 390.5 | 8.2 | HZE27L2 | 323.4 | 7.9 |
| HZE27B3 | 705.5 | 8.3 | HZE27L3 | 613.8 | 9.6 |
| HZM6B1 | 304.8 | 8.0 | HZM6L1 | 85.8 | 7.9 |
| HZM6B2 | 180.6 | 7.4 | HZM6L2 | 131.8 | 7.3 |
| HZM6B3 | 40.7 | 7.6 | HZM6L3 | 94.7 | 7.5 |
