# Supplementary material for: Skeletal Muscle Transcriptome Analysis of Hanzhong Ma Duck at Different Growth Stages Using RNA-Seq
Source: Biomolecules. 2021 Feb 19;11(2):315. doi: 10.3390/biom11020315 (PMC7927120; doi:10.3390/biom11020315)
Supplement: Supplementary file 1 [file biomolecules-11-00315-s001.zip › biomolecules-1104004-supplementary/Supplementary Materials/Table S3.docx]

**Table S3.** Statistics of sequence comparison between sample sequencing data and *Anas platyrhynchos* genome.

| **Samples** | **Total Reads** | **Mapped Reads** | **Uniq Mapped Reads** | **Multiple Map Reads** | **Reads Map to**  **'+'** | **Reads Map to**  **'-'** |
| --- | --- | --- | --- | --- | --- | --- |
| HZE17B1 | 43,524,534 | 34,692,789 (79.71%) | 30,557,989 (70.21%) | 4,134,800 (9.50%) | 14,532,292 (33.39%) | 16,408,872 (37.70%) |
| HZE17B2 | 54,789,896 | 45,025,987 (82.18%) | 39,542,047 (72.17%) | 5,483,940 (10.01%) | 18,451,754 (33.68%) | 21,179,628 (38.66%) |
| HZE17B3 | 58,324,696 | 47,148,208 (80.84%) | 41,661,109 (71.43%) | 5,487,099 (9.41%) | 19,623,497 (33.65%) | 22,277,888 (38.20%) |
| HZE17L1 | 54,959,678 | 44,188,252 (80.40%) | 38,767,223 (70.54%) | 5,421,029 (9.86%) | 18,220,992 (33.15%) | 20,813,537 (37.87%) |
| HZE17L2 | 55,472,750 | 44,961,166 (81.05%) | 39,853,430 (71.84%) | 5,107,736 (9.21%) | 19,021,295 (34.29%) | 21,351,849 (38.49%) |
| HZE17L3 | 48,698,420 | 39,416,885 (80.94%) | 34,841,755 (71.55%) | 4,575,130 (9.39%) | 16,470,145 (33.82%) | 18,650,257 (38.30%) |
| HZE21B1 | 52,841,414 | 42,267,314 (79.99%) | 37,061,546 (70.14%) | 5,205,768 (9.85%) | 17,575,756 (33.26%) | 20,006,654 (37.86%) |
| HZE21B2 | 56,195,314 | 44,335,025 (78.89%) | 39,767,940 (70.77%) | 4,567,085 (8.13%) | 19,529,844 (34.75%) | 21,321,243 (37.94%) |
| HZE21B3 | 55,178,342 | 43,577,992 (78.98%) | 37,530,665 (68.02%) | 6,047,327 (10.96%) | 17,233,178 (31.23%) | 20,302,428 (36.79%) |
| HZE21L1 | 53,487,930 | 44,028,664 (82.32%) | 37,086,572 (69.34%) | 6,942,092 (12.98%) | 16,379,640 (30.62%) | 20,181,828 (37.73%) |
| HZE21L2 | 44,608,336 | 36,269,751 (81.31%) | 30,056,409 (67.38%) | 6,213,342 (13.93%) | 12,741,500 (28.56%) | 16,381,518 (36.72%) |
| HZE21L3 | 59,867,386 | 48,999,024 (81.85%) | 40,761,721 (68.09%) | 8,237,303 (13.76%) | 17,385,436 (29.04%) | 22,158,110 (37.01%) |
| HZE27B1 | 61,201,624 | 47,287,468 (77.27%) | 40,587,670 (66.32%) | 6,699,798 (10.95%) | 18,844,108 (30.79%) | 22,090,781 (36.10%) |
| HZE27B2 | 51,139,538 | 39,001,466 (76.26%) | 34,419,233 (67.30%) | 4,582,233 (8.96%) | 16,576,118 (32.41%) | 18,616,132 (36.40%) |
| HZE27B3 | 55,588,028 | 41,661,537 (74.95%) | 36,733,017 (66.08%) | 4,928,520 (8.87%) | 17,818,132 (32.05%) | 19,913,788 (35.82%) |
| HZE27L1 | 53,548,116 | 44,965,068 (83.97%) | 28,981,166 (54.12%) | 15,983,902 (29.85%) | 6,787,142 (12.67%) | 17,217,025 (32.15%) |
| HZE27L2 | 54,294,482 | 44,186,500 (81.38%) | 29,271,975 (53.91%) | 14,914,525 (27.47%) | 7,474,924 (13.77%) | 17,074,461 (31.45%) |
| HZE27L3 | 54,270,992 | 43,292,754 (79.77%) | 28,241,302 (52.04%) | 15,051,452 (27.73%) | 7,396,939 (13.63%) | 16,798,626 (30.95%) |
| HZM6B1 | 41,896,388 | 30,008,951 (71.63%) | 25,100,513 (59.91%) | 4,908,438 (11.72%) | 12,579,341 (30.02%) | 14,423,415 (34.43%) |
| HZM6B2 | 57,357,736 | 42,085,752 (73.37%) | 34,520,667 (60.18%) | 7,565,085 (13.19%) | 16,544,663 (28.84%) | 19,753,185 (34.44%) |
| HZM6B3 | 44,135,118 | 33,253,436 (75.34%) | 27,720,533 (62.81%) | 5,532,903 (12.54%) | 13,175,912 (29.85%) | 15,575,693 (35.29%) |
| HZM6L1 | 50,381,348 | 39,041,038 (77.49%) | 29,472,522 (58.50%) | 9,568,516 (18.99%) | 11,885,005 (23.59%) | 17,179,720 (34.10%) |
| HZM6L2 | 54,497,530 | 44,052,872 (80.83%) | 31,336,511 (57.50%) | 12,716,361 (23.33%) | 9,958,385 (18.27%) | 18,059,674 (33.14%) |
| HZM6L3 | 53,126,970 | 41,100,544 (77.36%) | 30,857,647 (58.08%) | 10,242,897 (19.28%) | 11,632,204 (21.90%) | 17,679,210 (33.28%) |

**Note:** Total Reads: Reads number of Clean Data, not paired-end reads; **Mapped Reads:** Reads Number Mapped to the reference genome and the percentage in Clean Reads; **Unique Mapped Reads:** Reads Number Mapped uniquely mapped to the reference genome and the percentage in Clean Reads; **Multiple Mapped Reads:** Reads number multiplely mapped to reference genome and the percentage in Clean Reads; **Reads Map to '+':** Reads number mapped to the sense chain and the percentage in Clean Reads. **Reads Map to '-'**: Reads number mapped to the antisense chain and the percentage in Clean Reads.
