# Supplementary material for: Skeletal Muscle Transcriptome Analysis of Hanzhong Ma Duck at Different Growth Stages Using RNA-Seq
Source: Biomolecules. 2021 Feb 19;11(2):315. doi: 10.3390/biom11020315 (PMC7927120; doi:10.3390/biom11020315)
Supplement: Supplementary file 1 [file biomolecules-11-00315-s001.zip › biomolecules-1104004-supplementary/Supplementary Materials/Table S4.docx]

**Table S4.** SNPs from breast and leg muscle of Hanzhong Ma duck.

| **Sample** | **SNP Number** | **Genic SNP** | **Intergenic SNP** | **Transition** | **Transversion** | **Heterozygosity** |
| --- | --- | --- | --- | --- | --- | --- |
| HZE17B1 | 123,378 | 112,009 | 11,369 | 74.14% | 25.86% | 38.24% |
| HZE17B2 | 130,110 | 117,841 | 12,269 | 74.05% | 25.95% | 37.49% |
| HZE17B3 | 138,622 | 124,750 | 13,872 | 73.92% | 26.08% | 37.96% |
| HZE17L1 | 129,892 | 117,238 | 12,654 | 74.09% | 25.91% | 37.76% |
| HZE17L2 | 142,086 | 128,232 | 13,854 | 73.75% | 26.25% | 36.43% |
| HZE17L3 | 115,007 | 103,416 | 11,591 | 74.33% | 25.67% | 39.02% |
| HZE21B1 | 139,362 | 125,575 | 13,787 | 73.78% | 26.22% | 36.67% |
| HZE21B2 | 164,499 | 147,615 | 16,884 | 73.51% | 26.49% | 35.17% |
| HZE21B3 | 132,155 | 118,940 | 13,215 | 73.68% | 26.32% | 35.95% |
| HZE21L1 | 114,173 | 103,936 | 10,237 | 74.16% | 25.84% | 36.95% |
| HZE21L2 | 94,650 | 85,935 | 8,715 | 74.87% | 25.13% | 39.40% |
| HZE21L3 | 117,402 | 106,685 | 10,717 | 74.19% | 25.81% | 36.69% |
| HZE27B1 | 119,712 | 106,800 | 12,912 | 74.35% | 25.65% | 38.64% |
| HZE27B2 | 137,890 | 122,856 | 15,034 | 73.62% | 26.38% | 35.69% |
| HZE27B3 | 100,894 | 88,976 | 11,918 | 74.51% | 25.49% | 36.97% |
| HZE27L1 | 68,150 | 62,483 | 5667 | 75.74% | 24.26% | 40.96% |
| HZE27L2 | 86,513 | 78,016 | 8497 | 74.96% | 25.04% | 38.17% |
| HZE27L3 | 74,616 | 68,088 | 6528 | 75.74% | 24.26% | 41.34% |
| HZM6B1 | 56,327 | 51,586 | 4741 | 76.06% | 23.94% | 41.44% |
| HZM6B2 | 73,833 | 67,353 | 6480 | 75.16% | 24.84% | 39.47% |
| HZM6B3 | 69,820 | 64,154 | 5666 | 75.38% | 24.62% | 40.23% |
| HZM6L1 | 59,750 | 54,384 | 5366 | 75.91% | 24.09% | 40.87% |
| HZM6L2 | 82,939 | 75,594 | 7345 | 74.88% | 25.12% | 37.85% |
| HZM6L3 | 62,377 | 57,383 | 4994 | 75.84% | 24.16% | 40.49% |

**Note: Genic SNP**: total numbers of SNPs in the genic region; **Intergenic SNP:** total numbers of SNPs between genes; **Transition:** the percentage that the transition-type SNP accounts for all SNP locis; **Transversion:** the percentage that the transversion-type SNP loci accounts for all SNP sites; **Heterozygosity:** the percentage that the heterozygous SNPs account for all SNPs.
