# Supplementary material for: Skeletal Muscle Transcriptome Analysis of Hanzhong Ma Duck at Different Growth Stages Using RNA-Seq
Source: Biomolecules. 2021 Feb 19;11(2):315. doi: 10.3390/biom11020315 (PMC7927120; doi:10.3390/biom11020315)
Supplement: Supplementary file 1 [file biomolecules-11-00315-s001.zip › biomolecules-1104004-supplementary/Supplementary Materials/Table S5.docx]

**Table S5.** The most enriched GO terms related to muscle development.

| **Comparison group** | **The most enriched cellular components of GO terms related to muscle development** | | | | |
| --- | --- | --- | --- | --- | --- |
| HZE17B_vs_HZE21B | proteinaceous extracellular matrix | myosin complex | protein complex involved in cell adhesion | muscle tendon junction | cell junction |
| HZE21B_vs_HZE27B | myofibril | contractile fiber part | myosin complex | muscle tendon junction | myofilament |
| HZE27B_vs_HZM6B | myosin complex | striated muscle thin filament | actin filament bundle | MHC class I protein complex | contractile fiber |
| HZE17L_vs_HZE21L | myosin complex | myofibril | smooth muscle contractile fiber | muscle tendon junction | actin filament bundle |
| HZE21L_vs_HZE27L | proteinaceous extracellular matrix | myosin complex | striated muscle thin filament | muscle tendon junction | cell junction |
| HZE27L_vs_HZM6L | myosin complex | MHC class I protein complex | cell-cell junction | muscle tendon junction | extracellular matrix component |
|  | **The most enriched molecular function of GO terms related to muscle development** | | | | |
| HZE17B_vs_HZE21B | Extracellular matrix structural constituent | microtubule motor activity | muscle alpha-actinin binding | actin binding | transaminase activity |
| HZE21B_vs_HZE27B | microtubule motor activity | microtubule binding | muscle alpha-actinin binding | protein serine/threonine kinase activator activity | actin filament binding |
| HZE27B_vs_HZM6B | extracellular matrix structural constituent | actin filament binding | extracellular matrix binding | muscle alpha-actinin binding | microtubule motor activity |
| HZE17L_vs_HZE21L | extracellular matrix structural constituent | microtubule motor activity | muscle alpha-actinin binding | structural constituent of muscle | microtubule binding |
| HZE21L_vs_HZE27L | extracellular matrix structural constituent | muscle alpha-actinin binding | microtubule binding | microtubule motor activity | fibronectin binding |
| HZE27L_vs_HZM6L | extracellular matrix structural constituent | microtubule motor activity | muscle alpha-actinin binding | actin filament binding | motor activity |
|  | **The most enriched biological process of GO terms related to muscle development** | | | | |
| HZE17B_vs_HZE21B | mitotic cell cycle | negative regulation of skeletal muscle tissue development | regulation of skeletal muscle contraction | muscle cell cellular homeostasis | muscle organ morphogenesis |
| HZE21B_vs_HZE27B | positive regulation of protein kinase activity | negative regulation of striated muscle tissue development | muscle cell cellular homeostasis | Muscle filament sliding | muscle organ morphogenesis |
| HZE27B_vs_HZM6B | tendon development | myofibril assembly | transition between fast and slow fiber | muscle cell cellular homeostasis | muscle tissue development |
| HZE17L_vs_HZE21L | endodermal cell differentiation | embryonic skeletal system morphogenesis | negative regulation of striated muscle tissue development positive | regulation of G2/M transition of mitotic cell cycle | non-canonical Wnt signaling pathway via JNK cascade |
| HZE21L_vs_HZE27L | embryonic skeletal system morphogenesis | negative regulation of skeletal muscle tissue development | striated muscle cell proliferation | negative regulation of transforming growth factor-beta secretion | muscle system process |
| HZE27L_vs_HZM6L | tendon development | embryonic skeletal system morphogenesis | negative regulation of transforming growth factor-beta secretion | muscle cell cellular homeostasis | regulation of skeletal muscle contraction |
