# Supplementary material for: Skeletal Muscle Transcriptome Analysis of Hanzhong Ma Duck at Different Growth Stages Using RNA-Seq
Source: Biomolecules. 2021 Feb 19;11(2):315. doi: 10.3390/biom11020315 (PMC7927120; doi:10.3390/biom11020315)
Supplement: Supplementary file 1 [file biomolecules-11-00315-s001.zip › biomolecules-1104004-supplementary/Supplementary Materials/Table S6.docx]

**Table S6.** Top 5 in KEGG enrichment.

| **Comparison group** | **KEGG Enrichment** | | | | |
| --- | --- | --- | --- | --- | --- |
| HZE17B_vs_HZE21B | regulation of actin cytoskeleton | cell cycle | focal adhesion | purine metabolism | neuroactive ligand-receptor interaction |
| HZE21B_vs_HZE27B | oxidative phosphorylation | neuroactive ligand-receptor interaction | adrenergic signaling in cardiomyocytes | carbon metabolism | calcium signaling pathway |
| HZE27B_vs_HZM6B | oxidative phosphorylation | calcium signaling pathway | carbon metabolism | endocytosis | Insulin signaling pathway |
| HZE17L_vs_HZE21L | oxidative phosphorylation | focal adhesion | MAPK signaling pathway | ECM-receptor interaction | regulation of actin cytoskeleton |
| HZE21L_vs_HZE27L | oxidative phosphorylation | carbon metabolism | focal adhesion | calcium signaling pathway | MAPK signaling pathway |
| HZE27L_vs_HZM6L | focal adhesion | ECM-receptor interaction | calcium signaling pathway | regulation of actin cytoskeleton | neuroactive ligand-receptor interaction |
